# Novel Method for Rapid Detection of Spatiotemporal HIV Clusters Potentially Warranting Intervention

**DOI:** 10.3201/eid2505.180776

**Published:** 2019-05

**Authors:** Arthur G. Fitzmaurice, Laurie Linley, Chenhua Zhang, Meg Watson, Anne Marie France, Alexandra M. Oster

**Affiliations:** Centers for Disease Control and Prevention, Atlanta, Georgia, USA (A.G. Fitzmaurice, L. Linley, M. Watson, A.M. France, A.M. Oster);; ICF International, Atlanta (C. Zhang)

**Keywords:** HIV diagnosis, surveillance system, outbreak detection, spatiotemporal cluster, space–time, HIV, viruses, HIV/AIDS

## Abstract

Rapid detection of increases in HIV transmission enables targeted outbreak response efforts to reduce the number of new infections. We analyzed US HIV surveillance data and identified spatiotemporal clusters of diagnoses. This systematic method can help target timely investigations and preventive interventions for maximum public health benefit.

Despite innovations in HIV prevention and treatment, HIV outbreaks do occur in the United States. Local public health staff identified >200 persons with HIV resulting from an injection drug use (IDU)–associated outbreak in 2015 in Scott County, Indiana (*1*). The multipronged outbreak response included the establishment of Indiana’s first syringe services program. The number of cases might have been worse without intervention, suggesting the value of rapidly detecting and responding to increases in HIV transmission, whether related to IDU or other transmission modes.

The Centers for Disease Control and Prevention (CDC) recently began using HIV nucleotide sequence data from the National HIV Surveillance System (NHSS) to identify clusters of recent and rapid HIV transmission (*2*). Sequences are generated through HIV drug resistance testing routinely conducted as part of clinical care, but sequence reporting to health departments and CDC can be delayed or incomplete (*3*). Case surveillance data (i.e., reported diagnoses), which are timelier and more complete than sequence data, can be used to detect spatiotemporal increases in diagnoses.

CDC has not previously used systematic methods to analyze HIV case surveillance data to detect outbreaks as they occur. We developed a method to identify spatiotemporal clusters of increased diagnoses. Our proposed method enables efficient analysis at local and national levels to generate spatiotemporal alerts representing concentrated increases that require further investigation.

## The Study

We reviewed non–HIV outbreak detection literature and methods employed by disease and syndromic surveillance programs at CDC and in several state and local health departments. Methods generally inferred outbreaks from statistically significant increases above historical baselines (*4**–**6*). We tested analytic parameters on NHSS data to adapt existing methodologies. For example, HIV symptom onset and diagnosis can be delayed compared with other infectious diseases, so we varied frames for batching data and manually compared method outputs to determine optimal parameters based on epidemiologists’ assessments of the most concerning clusters. This systematic method detects increases in HIV diagnoses above expected baselines (i.e., alerts) in specified geographic areas. 

**Figure 1:**
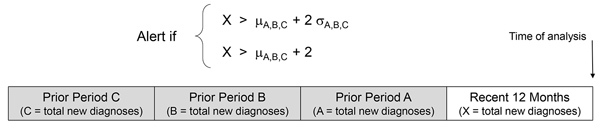
Alert criteria used in method for identifying spatiotemporal clusters of HIV diagnoses. For each cluster, the total number of cases (X) in a specified geographic area during the most recent 12 months exceeds the baseline mean (μ) of the previous 3 12-month periods by >2 SD (σ) and >2 diagnoses.

We applied this method to NHSS data reported from all 50 US states and the District of Columbia, examining the numbers of cases by state and county or county equivalent (e.g., borough, parish; hereafter, collectively referred to as “county” and including the District of Columbia). For each state or county, we determined the total number of diagnoses during the most recent 12 months (January–December 2016) on the basis of residence address at time of HIV diagnosis (Figure 1). We calculated the baseline as mean diagnoses in the 3 prior 12-month periods (calendar years 2013, 2014, and 2015). An alert was generated in a geographic area when the total number of cases during the most recent 12 months was >2 SD and >2 diagnoses greater than the baseline mean. The latter criterion eliminates alerts resulting from small diagnosis levels (e.g., baseline of 0 alerting with only 1 diagnosis). We repeated these analyses limiting to IDU-related diagnoses, excluding men who reported both male-to-male sexual contact and IDU.

State-level alerts occurred for 4 (8%) of 50 states (Midwest 3, South 1); county-level alerts occurred for 143 (5%) of 3,142 counties nationwide (Table). A median of 2 and mean of 4 counties per state had alerts. Using the exact Pearson test for homogeneity, we determined that alerting counties were disproportionately located in the Northeast (15%; p<0.001) and South (59%; p<0.001), compared with nonalerting counties in the Northeast (7%) and South (45%). Among cases with reported IDU risk, alerts occurred for 2 states in the Midwest, 1 state in the West, and 21 counties, which were located mostly in the South (38%) and Midwest (29%). Baseline rates for county-level IDU alerts averaged 0.3–9 diagnoses per year.

**Table:** Distribution of spatiotemporal clusters of HIV diagnoses among counties in 50 states and the District of Columbia, 2016

Characteristic	All diagnoses				Diagnoses attributable to injection drug use		
	Counties with alerts, no. (%)*	Counties without alerts, no. (%)	p value		Counties with alerts, no. (%)*	Counties without alerts, no. (%)	p value
Region (*7*)							
Northeast	21 (15)	196 (7)	0.0002		3 (14)	214 (7)	0.18
Midwest	27 (19)	1,028 (34)	0.0001		6 (29)	1,049 (34)	0.63
South	84 (59)	1,338 (45)	0.0009		8 (38)	1,414 (45)	0.51
West	11 (8)	437 (15)	0.022		4 (19)	444 (14)	0.53
Baseline mean annual HIV diagnoses, 2013–2015							
<3	52 (36)	2,128 (71)	<10^–4^		13 (62)	2,176 (69)	0.46
3–9	40 (28)	463 (15)	<10^–4^		8 (38)	495 (16)	0.0056
>9	51 (36)	408 (14)	<10^–4^		0	459 (15)	0.057
Total counties	143 (100)	2,999 (100)			21 (100)	3,121 (100)	

*An alert occurred when the number of diagnoses in 2016 increased by >2 SD and >2 diagnoses compared with the mean annual baseline over the preceding 3 years (2013–2015).

## Discussion

**Figure 2:**
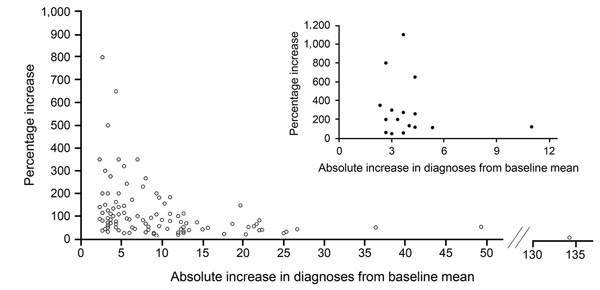
Percentage and absolute increases in annual HIV diagnoses above 3-year baseline means used in method for identifying spatiotemporal clusters of HIV diagnoses. Alerts are shown for 138 counties, as well as 21 county alerts attributable to injection drug use (inset). Five county alerts with 0 baseline diagnoses not shown (infinite percentage increase).

We aimed to develop a spatiotemporal cluster detection method that could efficiently be used and adapted to identify potential increases in HIV transmission in different local contexts. We identified significant increases in HIV diagnoses across all regions, capturing alerts from counties with small, medium, and large baseline numbers of HIV diagnoses. Some counties had small increases in the number of diagnoses and large percentage increases; others had larger increases in numbers but smaller increases in percentages (Figure 2). IDU-attributable diagnoses constitute a small proportion of total diagnoses, so the ability to identify potential IDU transmission clusters by analyzing IDU-attributable diagnoses separately is a strength of this method. Transmission through sexual and other risk networks might cross arbitrary geographic boundaries, but this method uses administrative boundaries aligned with existing data systems, so surveillance staff at state and local levels can automate monthly data analyses. States can conduct analyses at intermediary levels between state and county (e.g., regions within a state), and state or local health departments can analyze smaller areas (e.g., census tracts); national analyses will be vital for identifying spatiotemporal clusters across state boundaries.

We discussed our results with several state and local health departments that expressed interest in a robust, systematic method for routine identification of spatiotemporal clusters. They confirmed that this method identified alerts where they had recently begun responding and that new alerts provided actionable information regarding concerning HIV transmission increases.

Small median and mean numbers of alerts suggest reasonable investigative loads for this method. Batching data into moving 12-month frames reduces alerts resulting from seasonal variability and data noise. The chronic nature of HIV infection means that related cases might not be diagnosed until months or years after infection, so the 12-month analysis frame might not capture all related diagnoses, but it does account for delays between diagnosis and reporting to surveillance systems. These delays need to be addressed differently across states (*8*). State and local health departments with longer delays should improve reporting processes or analyze preliminary data; others can adapt the method by lagging or contracting the analysis frame.

Further investigation is needed to determine whether spatiotemporal clusters represent true increases in HIV transmission. Alerts might result from programmatic artifacts, although local epidemiologists would be aware of such programmatic influences (e.g., testing campaigns resulting in increased diagnoses not representing recent transmission). Reviewing testing history, partner services, contact tracing, and molecular data might help determine whether alerts represent clusters of recent infections that warrant investigation. Future evaluation will assess the extent to which this method identifies recent transmission and whether modifications might improve the method for different contexts.

The ideal cluster and outbreak detection system would use both case surveillance and molecular sequence-based approaches. Each method might help overcome the other’s limitations. Although some alerts occurred in counties with large baseline HIV numbers, this method is less sensitive for these areas and might not capture all meaningful clusters. Analysis of sequence data is crucial for identifying transmission clusters in areas with larger numbers of cases and those distributed over broader geographic areas. However, this method is timelier than molecular methods and can provide state and local health officials with actionable data for early investigation. This factor might be particularly necessary for identifying increases in transmission associated with IDU, given increasing opioid use and the potential for rapid spread of HIV among vulnerable populations (*1**,**9**–**11*). 

## Conclusions

In summary, we developed a systematic method to identify spatiotemporal clusters of HIV diagnoses. Routine use of this method in near real-time can automate detection of increases in HIV diagnoses meriting further investigation, helping state and local health departments prioritize and target HIV prevention and outbreak response efforts for maximum public health benefit.
